# Effects of h-BN Doping on the Microstructure, Mechanical Properties, and Dielectric Properties of Silicon Nitride Ceramics

**DOI:** 10.3390/ma19132775

**Published:** 2026-06-30

**Authors:** Xia Liu, Ying Wang, Hongfei Shao, Xin Zhang, Jinyong Zhang

**Affiliations:** 1 Institute of Nonmetallic Materials, Jinan 250061, China; liuxia2001@163.com (X.L.); shaohongfei@163.com (H.S.); z1228802607@163.com (X.Z.); 2 State Key Laboratory of Advanced Technology for Materials Synthesis Processing, Wuhan University of Technology, Wuhan 430070, China; 13842953586@163.com

**Keywords:** Si_3_N_4_/BN composite ceramic, spark plasma sintering, mechanical properties, dielectric properties

## Abstract

Silicon nitride ceramics exhibit excellent structural strength and electromagnetic wave transmission performance, yet demonstrate significant thermal shock instability under extreme conditions. Boron nitride (BN), on the other hand, possesses outstanding thermal shock resistance and electromagnetic wave transmission properties but exhibits relatively lower structural strength. Compositing these two materials holds promise for developing an integrated material that combines high-temperature load-bearing capacity with wave transmission capability. This study employed spark plasma sintering (SPS) technology to systematically investigate how varying BN content affects the sintering densification process and microstructural evolution of Si_3_N_4_/BN composite ceramics. Furthermore, we elucidated the mechanisms by which material composition and processing parameters influence key mechanical properties, dielectric characteristics, and other multifunctional attributes of the composites, providing a theoretical foundation for synergistic optimization design. The results indicate that BN incorporation suppresses both the phase transition from α-Si_3_N_4_ to β-Si_3_N_4_ during sintering and the growth of elongated β-Si_3_N_4_ crystals: the former hinders densification while the latter promotes it, resulting in a dual competitive mechanism that initially increases followed by decreases in sintered density. The effects of BN content on elastic modulus and fracture toughness align with trends in sintering density, whereas hardness, flexural strength, dielectric constant, and dielectric loss all show a monotonically decreasing trend with increasing BN content.

## 1. Introduction

Extreme service environments impose stringent demands on material performance. Single-phase ceramic materials often face limitations due to their inherent properties, making them inadequate for practical applications. Taking Si_3_N_4_ ceramics as an example, they exhibit excellent strength, high-temperature stability, and superior dielectric properties, offering broad application potential in structure-functional integrated ceramic components. However, under extreme thermal shock conditions such as rapid temperature changes, their thermal shock resistance frequently fails to meet engineering requirements, thereby restricting their use in high-temperature rapid thermal shock environments [1,2,3]. Compared to Si_3_N_4_, BN demonstrates relatively lower strength, hardness, and elastic modulus but exhibits superior thermal conductivity, dielectric properties, and machinability. According to composite material design principles, incorporating Si_3_N_4_ into a BN matrix significantly enhances the mechanical properties of multiphase ceramics; conversely, adding BN to a Si_3_N_4_ matrix markedly improves both thermal shock resistance and machinability while optimizing dielectric and wave transmission characteristics, paving the way for BN-based ceramics as integrated materials combining structural integrity with electromagnetic wave transmission capabilities under high-temperature rapid thermal shock conditions [4]. It should be noted that comprehensive performance optimization of such multiphase ceramics relies not only on simple phase mixing but also closely depends on the spatial distribution of component phases. Phase composition ratios and preparation processes fundamentally influence sintering behavior, final microstructure, and ultimately determine material performance under service conditions.

Gong et al. [5] prepared Si_3_N_4_/BN multiphase ceramics using hot-press sintering. Their study revealed that the introduction of BN hindered the sintering densification of the Si_3_N_4_ matrix, necessitating the use of sintering additives to form a liquid phase at high temperatures to facilitate the transformation of silicon nitride from the α-phase to the β-phase. When Al_2_O_3_ and Y_2_O_3_ were employed as sintering additives, significant amounts of β-Si_3_N_4_ were formed in the sintered sample, thereby enhancing its flexural strength. Li et al. [6] replaced hot-press sintering with discharge plasma sintering (SPS), demonstrating that this method not only accelerates sintering but also achieves excellent densification and uniform microstructure within a short time, resulting in samples with higher flexural strength. An et al. [7] further found that Si_3_N_4_/BN multiphase ceramics prepared by SPS exhibit insensitivity to sintering pressure within the high-density range; increasing pressure beyond 40 MPa did not significantly improve material properties. Zou, W. et al. [4] investigated the effect of increasing BN content on the processability of multiphase ceramics using a hot-press sintering system, revealing that while higher BN concentrations reduced material density, hardness, and flexural strength, they markedly improved processability. Jin et al. [8] prepared Si_3_N_4_/BN multiphase ceramics via atmosphere-pressure sintering and found that when the BN content was 30%, the dielectric constant of the composite could be optimized to 4, but its room-temperature flexural strength decreased to 160 MPa. Although BN effectively reduced the dielectric constant of Si_3_N_4_ ceramics, it significantly hindered the densification process of the multiphase ceramics during GPS sintering. This indicates that both material composition and sintering conditions require further optimization.

To this end, this study employed discharge plasma sintering technology to systematically investigate the effects of BN addition on the sintering behavior, microstructure, mechanical properties, and dielectric performance of Si_3_N_4_/BN multiphase ceramics by precisely controlling the BN content in silicon nitride-based multiphase ceramics, aiming to provide technical support for developing such structure-wave-guiding integrated materials.

## 2. Experiment

### 2.1. Raw Materials

**Figure 1:**
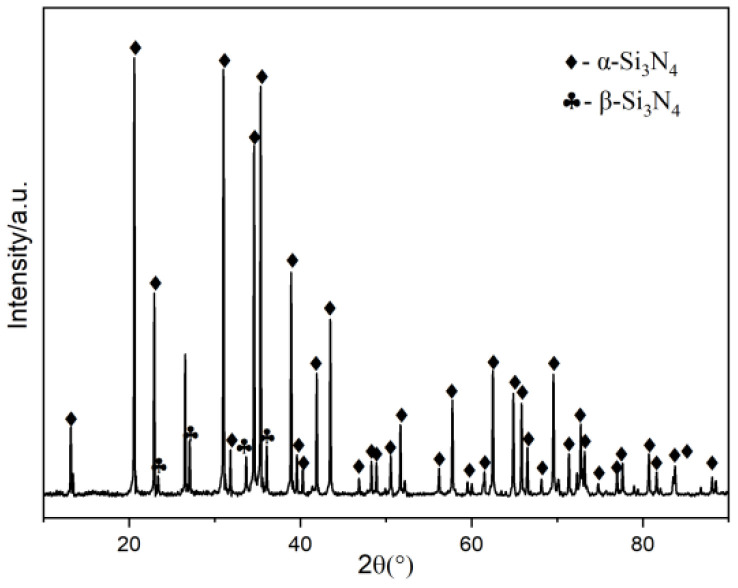
XRD analysis results of Si_3_N_4_ powder.

The powder materials used in the experiment were purchased from Afasha Co., Ltd. (Ryugasaki-shi, Japan). The Si_3_N_4_ powder had a purity of 99.9% and an average particle size of approximately 4 μm. Phase analysis revealed that the powder was predominantly composed of α-Si_3_N_4_ (with a content exceeding 94%) and contained trace amounts of β-Si_3_N_4_ (as shown in Figure 1 of the XRD spectrum). The BN raw powder exhibited a purity exceeding 99%, with its main phase being hexagonal boron nitride (h-BN) and an average particle size of about 0.5 μm. Additionally, 3 wt% Al_2_O_3_ and 5 wt% Y_2_O_3_ were employed as sintering aids.

### 2.2. Experimental Methods

This experiment employed Si_3_N_4_ powder as the matrix, with BN powders added at concentrations of 5 wt%, 10 wt%, 20 wt%, and 30 wt% as reinforcement phases. Additionally, Al_2_O_3_ (equivalent to 3% of the total mass of the mixed powder) and Y_2_O_3_ (equivalent to 5%) were incorporated as sintering aids. The mixture was subjected to wet ball milling for 12 h using ethanol as the dispersing medium and agate balls as the grinding media, with a ball-to-material ratio of 1:2. The resulting slurry was then transferred to a rotary evaporator to remove ethanol and dried in a vacuum drying oven at 60 °C for 24 h. The dried powder was sieved through a 200-mesh sieve.

Subsequently, the resulting composite powder was subjected to sintering in a discharge plasma sintering furnace. The sintering process was conducted under an argon protective atmosphere at a heating rate of 100 °C/min, under a pressure of 50 MPa, and maintained for 5 min. The sintered samples were then cut, ground, and polished to prepare standard specimens suitable for structural characterization, mechanical property analysis, and dielectric property testing. For clarity, the sintered samples were designated SN5, SN10, SN20, and SN30 based on the BN addition levels; the pure Si_3_N_4_ sample without BN was designated SN0 as the control group.

### 2.3. Testing and Characterization

The density of sintered samples was determined using the Archimedes displacement method with distilled water as the medium. The Vickers hardness was measured with a Vickers hardness tester (Model 430 SVD; Wolpert Wilson Measuring Instruments Co., Ltd., Shanghai, China) under a loading force of 9.8 N for a holding time of 15 s. The flexural strength was tested on a universal testing machine (MTS810, MTS, Eden Prairie, MN, USA) using a sample dimensions of 3 mm × 4 mm × 36 mm with a span of 30 mm at a loading rate of 0.5 mm/min. Fracture toughness (KIC) was measured by the single-edge notch beam method on the same instrument, employing a notch width of 0.2 mm and depth of 2 mm. The material’s phase composition was analyzed qualitatively and quantitatively using an X-ray diffractometer (XRD, Rigaku Ultima III, Tokyo, Japan) with a monochromatic Cu-Kα radiation source (wavelength 0.1540598 nm, operating voltage 40 kV, current 40 mA) and a scanning range of 10–90°. The microstructure of the samples was observed via scanning electron microscopy (SEM, Hitachi 3400, Tokyo, Japan). To clearly visualize the grain structure, the samples were etched with molten NaOH at 400 °C for 2 min. The dielectric constant and dielectric loss tangent of the samples at room temperature were measured using the high-Q cavity method on specimens measuring ϕ 18 mm × 1 mm, within a frequency range of 20 GHz to 35 GHz.

## 3. Results and Discussion

### 3.1. Sintering Densification Behavior of Si_3_N_4_/BN Composite Powder

**Figure 2:**
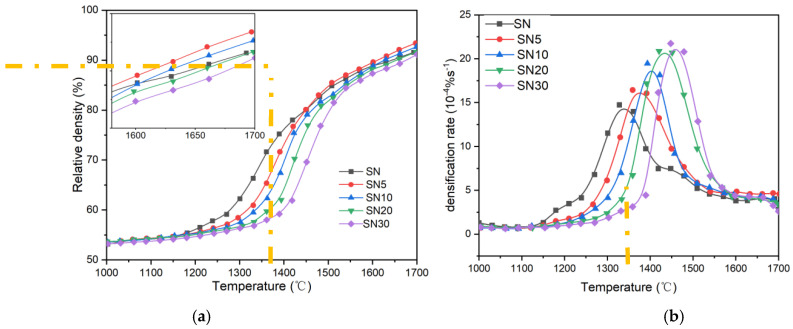
SPS sintering densification curves and densification rate curves for samples with different BN contents: (**a**) curve of relative density versus temperature; (**b**) curve of densification rate versus temperature.

Figure 2 illustrates the densification curves and corresponding densification rate curves of Si_3_N_4_/BN composites with varying BN contents during discharge plasma sintering (SPS) under constant heating rate and pressure conditions. As shown in Figure 2a, the relative density of all samples remained consistent throughout both the initial and final stages of sintering, showing no significant variation with changes in BN content. However, during the intermediate sintering phase, a pronounced lag in densification progress was observed as BN content increased. This phenomenon is further evident in the densification rate curve (Figure 2b): the peak densification rate occurred at a significantly delayed time point and required substantially higher temperatures. Notably, the maximum densification rate increased with rising BN content, indicating that BN does not merely inhibit the sintering process. At temperatures below 1300 °C, BN addition markedly reduced the densification rate of the mixed powders; however, above 1300–1400 °C, pure Si_3_N_4_ (SN) samples exhibited lower densification rates than all BN-containing samples, with higher BN content correlating with faster densification rates. At 1600 °C, the relative density ranged between 85% and 90%, indicating that interconnected pores within the green body had largely disappeared and the sintering had entered its advanced stage [9]. At this stage, all samples exhibited similar densities, and the effect of relative density on densification rate was largely consistent; however, BN-containing composite samples maintained a higher densification rate than pure SN samples.

It is generally believed that the densification during the early stage of Si_3_N_4_ ceramic sintering is primarily driven by plastic deformation and particle rearrangement mechanisms at high temperatures, while the later stage is mainly governed by the phase transition from α-Si_3_N_4_ to β-Si_3_N_4_. When the temperature exceeds 1300–1400 °C, the α-Si_3_N_4_ in the raw material dissolves under the action of sintering additives, after which thermodynamically stable β-Si_3_N_4_ precipitates and grows from the liquid phase [10].

Given that the pressure remained constant throughout the sintering process in this experiment, BN’s layered structure makes it resistant to compression and reorganization, and both BN and Si_3_N_4_ exhibit extremely low diffusion coefficients at high temperatures, it is inferred that the increased densification rate during the mid-to-late sintering stage as BN content rises may be closely linked to BN’s phase transition relative to Si_3_N_4_ and its crystal growth kinetics.

### 3.2. Structural Evolution During Sintering of Si_3_N_4_/BN Composite Powder

**Figure 3:**
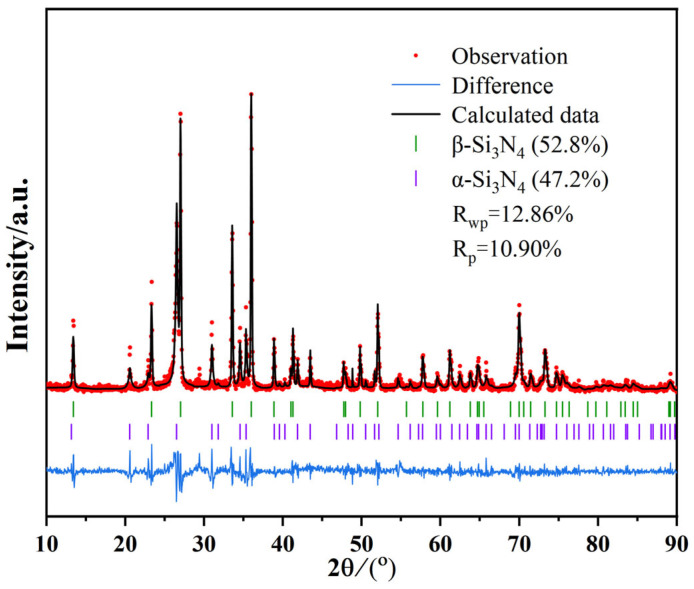
XRD morphology of silicon nitride/BN composites with different BN contents.

Figure 3 shows the X-ray diffraction spectra of Si_3_N_4_/BN composite ceramic samples at different BN mass fractions. The detected phases primarily include α-Si_3_N_4_, β-Si_3_N_4_, and BN. Due to the low BN content, the diffraction peak intensity is relatively weak and thus not prominent in the XRD patterns. The newly formed β-Si_3_N_4_ phase mainly originates from a phase transition of α-Si_3_N_4_ during sintering.

As previously mentioned, the introduction of BN may influence the phase transition process. To investigate this, our study employed specialized software to identify characteristic peaks corresponding to the [102] and [210] crystal planes of Si_3_N_4_ and analyzed the extent of phase transformation in α-Si_3_N_4_ across different samples [4]. The calculated relative contents of α-Si_3_N_4_ and β-Si_3_N_4_ phases in each sample are presented in Table 1. Results demonstrate that all samples exhibited a phase transition from α-Si_3_N_4_ to β-Si_3_N_4_. Specifically, samples SN0 and SN5 showed complete conversion of α-Si_3_N_4_ to β-Si_3_N_4_; when BN content increased to 10 wt%, the residual α-Si_3_N_4_ content remained at 18.2 wt%. Further increases in BN content resulted in unconverted α-Si_3_N_4_ levels of 35.1 wt% and 54.6 wt% in sample SN20, respectively. This clearly indicates that elevated BN content inhibits the transformation from α-Si_3_N_4_ to β-Si_3_N_4_. Notably, while material density decreased with increasing BN content in all BN-added samples, SN5 and SN10 samples exhibited significantly higher densities compared to SN0. Given the positive correlation between Si_3_N_4_’s phase transition and densification behavior, BN likely exerts an underlying yet fully elucidated promoting effect on densification.

**Figure 4:**
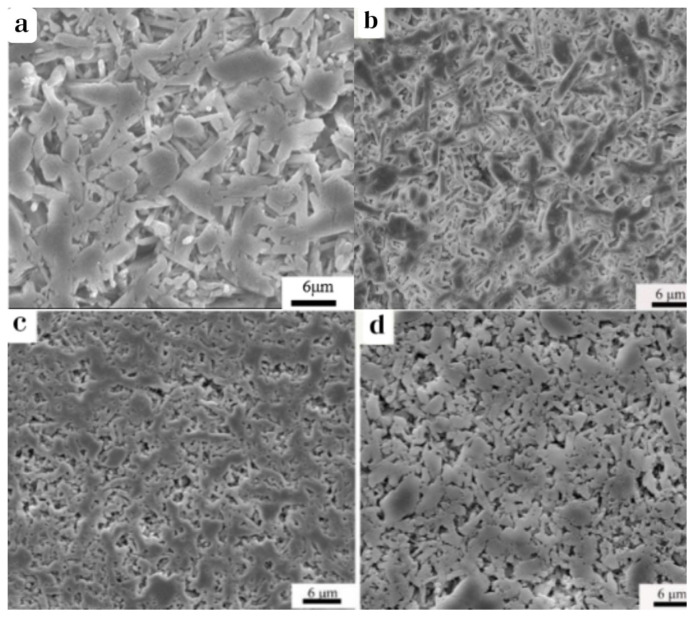
Microstructural changes of Si_3_N_4_/BN composite ceramics under different BN content: (**a**) SN0; (**b**) SN5; (**c**) SN10; (**d**) SN20.

Figure 4 presents SEM analysis results of polishing and etching profiles for Si_3_N_4_/BN multiphase ceramics with varying BN content. The elongated columnar grains depicted correspond to β-Si_3_N_4_, while the equiaxed fine particles represent α-Si_3_N_4_, with some flaky grains constituting the BN phase. Fine α-Si_3_N_4_ and BN grains are uniformly distributed within the interstices of the elongated β-Si_3_N_4_ crystals. The figure demonstrates that as BN content increases, significant changes occur in β-Si_3_N_4_ grain morphology: the introduction of BN phase reduces β-Si_3_N_4_ content and leads to decreases in both grain size and aspect ratio. BN addition not only influences the phase transformation process of Si_3_N_4_ but also inhibits the growth of elongated β-Si_3_N_4_ crystals. Given that randomly arranged columnar structures tend to form voids difficult to fill during sintering, this inhibition mechanism partially enhances sintering density. In summary, the relative density of Si_3_N_4_/BN multiphase ceramics exhibits an initial increase followed by a decrease with rising BN content, resulting from the combined effects of phase transformation suppression and grain growth inhibition mechanisms.

### 3.3. Properties of Si_3_N_4_/BN Composite Ceramics

The dielectric and mechanical properties of Si_3_N_4_/BN composite ceramics are closely related to their microstructure, though the influence mechanisms vary among these properties [11,12]. In this section, we conducted experimental analysis on the typical mechanical and dielectric properties of composite ceramic samples with different BN contents prepared under the aforementioned conditions.

**Figure 5:**
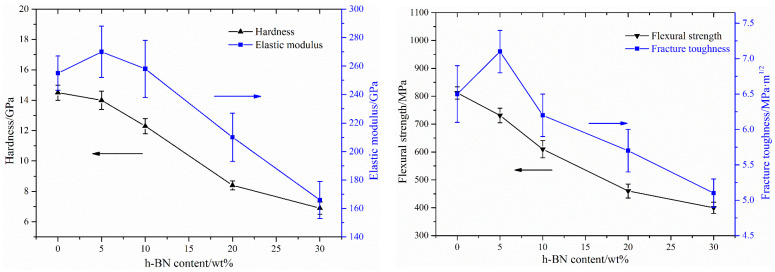
Effect of BN content on the typical mechanical properties of Si_3_N_4_/BN composite ceramics.

Figure 5 illustrates the variation patterns of typical mechanical properties of Si_3_N_4_/BN composite ceramics under different BN content levels. Analysis reveals that both hardness and flexural strength of the composites exhibit a monotonically decreasing trend with increasing BN content. When the BN content reaches 30%, the Vickers hardness of Si_3_N_4_/BN composites decreases from 14.5 GPa to 6.9 GPa, while flexural strength drops from 812 ± 22 MPa to 416 MPa. However, the elastic modulus and fracture toughness of these composites follow similar trends to their relative density, showing initial increases followed by declines. The SN5 sample demonstrates the highest elastic modulus (272 GPa) and fracture toughness (7.1 MPa·m^−1/2^), representing improvements of approximately 7% and 9% compared to SN0 samples. Given that pure Si_3_N_4_ exhibits the highest hardness while SN5, SN10, and SN20 samples all have higher relative densities than pure Si_3_N_4_, the monotonic decrease in hardness and flexural strength may be attributed to the presence of the weak-phase BN, lower β-Si_3_N_4_ grain content, and smaller aspect ratios. Extensive studies have demonstrated that the interlocking structure of β-Si_3_N_4_ grains is critical for achieving high flexural strength, whereas porosity has a lesser impact on mechanical properties compared to other isometric ceramic systems [13,14,15]. According to conventional composite material theory, elastic modulus primarily depends on component properties and volume fractions; treating pores as one component provides a reasonable explanation for the initial increase followed by decrease in modulus behavior. The variation in fracture toughness is influenced not only by the aforementioned factors but may also stem from the adsorption effect of the weak-phase BN and pores on crack propagation, as well as the associated fracture energy dissipation mechanisms; the precise mechanisms require further in-depth investigation.

**Figure 6:**
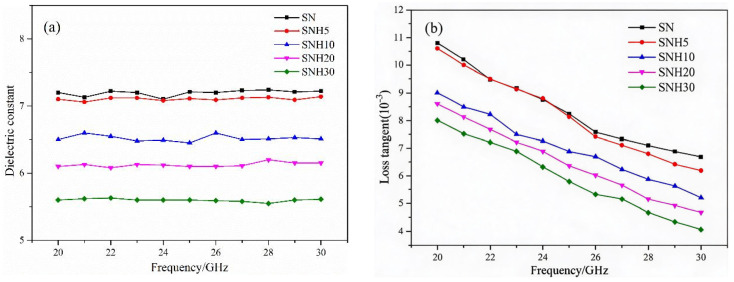
Dielectric constant and dielectric loss tangent of multiphase ceramic samples with different BN contents: (**a**) The variation law of dielectric constant of multiphase ceramic samples with different BN addition amounts in a specific frequency range; (**b**) The variation curve of dielectric loss tangent of the same sample at different frequencies.

Figure 6 illustrates the dielectric constant and dielectric loss tangent (tan δ) of Si_3_N_4_/BN composite ceramic samples with varying BN content. Observations reveal that all samples exhibited excellent stability in dielectric constant across the tested frequency range, showing minimal variation with frequency. At identical test frequencies, the dielectric constant decreased with increasing BN content, attributed to BN’s significantly lower dielectric constant (4.5) compared to Si_3_N_4_’s (approximately 7.2). The dielectric properties of composites can generally be predicted using simple mixing rules; thus, introducing BN—with its low dielectric constant—into the Si_3_N_4_ matrix correspondingly reduced the composite’s dielectric constant. Due to negligible differences in porosity, this effect was also insignificant. The influence of BN content on dielectric loss tangent (tan δ) mirrored its impact on dielectric constant: increased BN content led to more pores in the composite structure and higher amounts of non-phase-transiting α-Si_3_N_4_ phases, both contributing to reduced system dielectric constants and losses. Notably, all samples demonstrated higher dielectric constants and dielectric loss tangents—particularly the latter—than reported in the literature, likely attributable to high current application and enhanced carbon atmosphere during SPS sintering.

## 4. Conclusions

In this study, a series of Si_3_N_4_/BN composite ceramic samples were prepared using the discharge plasma sintering (SPS) technique, and the effects of BN content on the microstructure and mechanical properties of the composites were systematically investigated. The results demonstrate that BN effectively suppresses the phase transformation from α-Si_3_N_4_ to β-Si_3_N_4_ during sintering as well as the growth of elongated β-Si_3_N_4_ crystal grains. By exerting both promoting and inhibiting effects through distinct mechanisms, BN influences the sintering densification process, resulting in a trend where sinter density first increases followed by decrease. The impact of BN content on elastic modulus and fracture toughness aligns with the changes in sinter density; whereas hardness, flexural strength, dielectric constant, and dielectric loss all exhibit a monotonically decreasing trend with increasing BN content. These distinct behavioral patterns provide a theoretical foundation for the synergistic optimization of multiple performance parameters in Si_3_N_4_/BN composite ceramics.

## Figures and Tables

**Table 1:** Relative contents of α-phase and β-phase Si_3_N_4_ in Si_3_N_4_/BN samples with different BN concentrations.

	SN	SNH5	SNH10	SNH20	SNH30
α-Si_3_N_4_	0	0	19.8	35.1	54.6
β-Si_3_N_4_	100	100	80.2	64.9	45.4

## Data Availability

The original contributions presented in this study are included in the article. Further inquiries can be directed to the corresponding author.
